# Invasive Cancer Incidence — Puerto Rico, 2007–2011

**Published:** 2015-04-17

**Authors:** Mary Elizabeth O’Neil, S. Jane Henley, Simple D. Singh, Reda J. Wilson, Karen J. Ortiz-Ortiz, Naydi Pérez Ríos, Carlos R. Torres Cintrón, Guillermo Tortolero Luna, Diego E. Zavala Zegarra, A. Blythe Ryerson

**Affiliations:** 1 Division of Cancer Prevention and Control, National Center for Chronic Disease Prevention and Health Promotion, CDC; 2 Puerto Rico Central Cancer Registry.

Cancer is a leading cause of morbidity and death in Puerto Rico (1). To set a baseline for identifying new trends and patterns of cancer incidence, Puerto Rico Central Cancer Registry staff and CDC analyzed data from Puerto Rico included in U.S. Cancer Statistics (USCS) for 2007–2011, the most recent data available. This is the first report of invasive cancer incidence rates for 2007–2011 among Puerto Rican residents by sex, age, cancer site, and municipality. Cancer incidence rates in Puerto Rico were compared with those in the U.S. population for 2011. A total of 68,312 invasive cancers were diagnosed and reported in Puerto Rico during 2007–2011. The average annual incidence rate was 330 cases per 100,000 persons. The cancer sites with the highest cancer incidence rates included prostate (152), female breast (84), and colon and rectum (43). Cancer incidence rates varied by municipality, particularly for prostate, lung and bronchus, and colon and rectum cancers. In 2011, cancer incidence rates in Puerto Rico were lower for all cancer sites and lung and bronchus, but higher for prostate and thyroid cancers, compared with rates within the U.S. population. Identifying these variations can aid evaluation of factors associated with high incidence, such as cancer screening practices, and development of targeted cancer prevention and control efforts. Public health professionals can monitor cancer incidence trends and use these findings to evaluate the impact of prevention efforts, such as legislation prohibiting tobacco use in the workplace and public places and the Puerto Rico Cessation Quitline (2) in decreasing lung and other tobacco-related cancers.

Data on new cases of invasive cancer* diagnosed during 2007–2011 were abstracted from medical records at health-care facilities, including hospitals, physician’s offices, and pathology laboratories, following the North American Association of Central Cancer Registries data standards (3). The USCS dataset includes incidence data from CDC’s National Program of Cancer Registries and the National Cancer Institute’s Surveillance, Epidemiology, and End Results program (3,4). The National Program of Cancer Registries incidence data in this report were reported to the CDC as of November 30, 2013, and are the most recent available data.

Completeness of case ascertainment is one of six USCS publication criteria† (3,4). It is estimated using North American Association of Central Cancer Registries’ completeness algorithm (3), which is based on comparing observed cancer incidence and death rates with expected rates.§ A variation on this algorithm was used to derive the completeness of case ascertainment in Puerto Rico because of differences in population attributes: expected rates were based on U.S. Hispanic data only rather than on expected rates for the total U.S. population.

Incident cases were classified by anatomic site using the *International Classification of Diseases for Oncology, Third Edition* (ICD-O-3). Cases with hematopoietic histologies were further classified using the *WHO Classification of Tumours of Haematopoietic and Lymphoid Tissues, Fourth Edition*. Denominators for Puerto Rico’s incidence rates were sex-specific population estimates for Puerto Rico from the 2010 U.S. Census;¶ denominators used to calculate Puerto Rico municipality incidence rates were sex- and municipality-specific population estimates provided by the U.S. Census Bureau.** Annual incidence rates per 100,000 population were age-adjusted by the direct method to the 2000 U.S. standard population using 19 age-categories. When <16 cases were reported, the number and rate are not presented because of the potential for statistically unreliable estimates and the need to protect confidentiality (3).

Incidence rates of selected cancers over a 5-year period (2007–2011) were calculated for Puerto Rico. The incidence rates in 2011 of selected cancers in Puerto Rico and the United States (total population and by racial and ethnic groups) were compared. All central cancer registries included in the U.S. comparison met the USCS publication criteria for 2011, representing 99% coverage of the U.S. population (3). Maps were created using ArcGIS by rank-ordering the Puerto Rico municipalities’ incidence rates and then grouping into quartiles.

From 2007 to 2011, a total of 68,312 invasive cancers were diagnosed in Puerto Rico, approximately 13,662 invasive cases per year. The average annual age-adjusted incidence rate was 330 cases per 100,000 persons over the 5-year period. Age-adjusted incidence rates were higher among males (395 per 100,000) than among females (281 per 100,000) (Table). By age group, rates per 100,000 population during 2007–2011 were 14 among persons aged 0–19 years, 128 among those aged 20–49 years, 594 among those aged 50–64 years, 1,281 among those aged 65–74 years, and 1,597 among those aged ≥75 years (Table).

By cancer site, average annual rates were highest for cancers of the prostate (152 per 100,000 men), female breast (84 per 100,000 women), and colon and rectum (43 overall, 53 among men, and 35 among women) (Table). These three sites combined accounted for approximately half of cancers diagnosed between 2007 and 2011 (Table). Among men, the first, second, and third most common cancers were prostate, colon and rectum, and lung and bronchus (rates of 152, 53, and 25 per 100,000 men, respectively), while among women the leading sites were breast, colon and rectum, and thyroid (rates of 84, 35, and 29 per 100,000 women, respectively).

In 2011, Puerto Rico had a lower age-adjusted all-sites cancer incidence rate (339 per 100,000) than the United States (451), regardless of U.S. racial or ethnic group (467 for U.S. non-Hispanic blacks, 462 for U.S. non-Hispanic whites, and 351 for U.S. Hispanics) (data not shown). Prostate cancer incidence was higher in Puerto Rico (150 per 100,000 men) than the U.S. overall (128) and the U.S. Hispanic population (104), but lower than the U.S. non-Hispanic black population (198) (Figure 1). Breast cancer incidence in Puerto Rico (93 per 100,000 women) was similar to the U.S. Hispanic incidence (92), both of which were lower than the U.S. overall population (122). Lung and bronchus cancer incidence in Puerto Rico was lower than in the U.S. overall population (17 versus 61 per 100,000). Colon and rectum cancer incidence in Puerto Rico (43 per 100,000) was similar to the U.S. overall population (40), regardless of race or ethnicity (Figure 1). Thyroid cancer incidence in Puerto Rico (21 per 100,000) was higher than in U.S. non-Hispanic white (15), U.S. Hispanic (12), and U.S. non-Hispanic black populations (9) (Figure 1).

Prostate cancer had notably higher incidence rates in the southeastern municipalities than in the west; colon and rectum cancer appeared to be more commonly diagnosed in the south and west (Figure 2). Lung cancers were prominent in the eastern and central municipalities; female breast cancer rates are highest among many coastline municipalities. Thyroid cancer incidence rates were highest in the north-central region of Puerto Rico (Figure 2).

## Discussion

The Puerto Rico Central Cancer Registry has been collecting data on cancer in Puerto Rico since 1951 and has been part of National Program of Cancer Registries since 1997. This is the first report of the USCS dataset with the Puerto Rico cancer registry data and it shows that for 2011, the latest year for which data are available for comparison, the overall cancer incidence rate in Puerto Rico was lower than in the U.S. population. Puerto Rico had a lower rate of female breast cancer compared with U.S. non-Hispanic whites and blacks and the lowest rate of lung cancer compared with all race and ethnic groups included in this analysis. However, Puerto Rico had the second highest prostate cancer incidence rates after U.S. non-Hispanic blacks and it also had the highest incidence rate of thyroid cancer. Puerto Rico had similar incidence rates to U.S. populations for colon and rectum cancer.

Differences in reported cancer incidence rates between U.S. and Puerto Rican residents might be partly explained by differences in the prevalence of risk factors such as behaviors associated with cancers or in the use of cancer screening tests. Lower rates of female breast cancer incidence might be attributable to the protective effect of young age at first live birth, which is more common in Puerto Rico than in the United States (5). Also, CDC’s Behavioral Risk Factor Surveillance System data show that the prevalence of current cigarette smoking in Puerto Rico is low compared with U.S. states; only Utah is lower (6). Consistent with these data, National Health Interview Survey data show that U.S. Hispanics’ current cigarette smoking rate is generally lower than the rate in the general U.S. population (7), which might explain the lower rates of lung cancer.

What is already known on this topic?

As of 2012, cancer is a leading cause of illness and death in Puerto Rico. Many cancers are preventable.

What is added by this report?

Data on cancer incidence in Puerto Rico are now included in U.S. Cancer Statistics and show that during 2007–2011, the overall, age-adjusted, annual cancer incidence rate was 330 cases per 100,000 persons and varies by municipality. Cancer sites with the highest incidence included prostate (152), female breast (84), and colon and rectum (43). In 2011, overall, age-adjusted, annual cancer incidence in Puerto Rico was 339 cases per 100,000 persons compared with 451 in the United States, and incidence rates in Puerto Rico were lower for lung and bronchus cancer but higher for prostate and thyroid cancers.

What are the implications for public health practice?

Differing rates of cancer by municipality indicate a need to assess geographic variations in risk factor prevalence and cancer screening practices. The cancer rates for 2007–2011 will be critical for assessing the effectiveness of cancer prevention programs.

There are also geographic variations in cancer incidence by cancer site. As has been shown in previous investigations in Puerto Rico, the incidence rates of cancers of the kidney, pancreas, prostate, breast, colon and rectum, thyroid, and lung were higher in areas of Puerto Rico with higher socioeconomic position (8). However, prostate cancer incidence was also found to be high in the southeastern portion of the country, an area with a lower socioeconomic position. Possible explanations include higher rates of prostate-specific antigen testing in this region (9).

The findings in this report are subject to at least two limitations. First, delays in cancer reporting can result in an undercount of cancer incidence, particularly for the most recent years (10). Second, the 2011 population was estimated from the 2010 U.S. Census, which might lead to under- or over-estimations of incidence rates.

Data from population-based central cancer registries are important for monitoring trends over time and identifying opportunities to reduce cancer incidence and mortality, particularly among high-risk groups and underserved areas (3). Data from the Puerto Rico Central Cancer Registry are used to identify and select cancer control priorities, identify populations of interest for implementation of cancer control strategies††, and respond to concerns about possible cancer clusters on the island, as well as for evaluation of the impact of cancer control strategies (2).

## Figures and Tables

**FIGURE 1:**
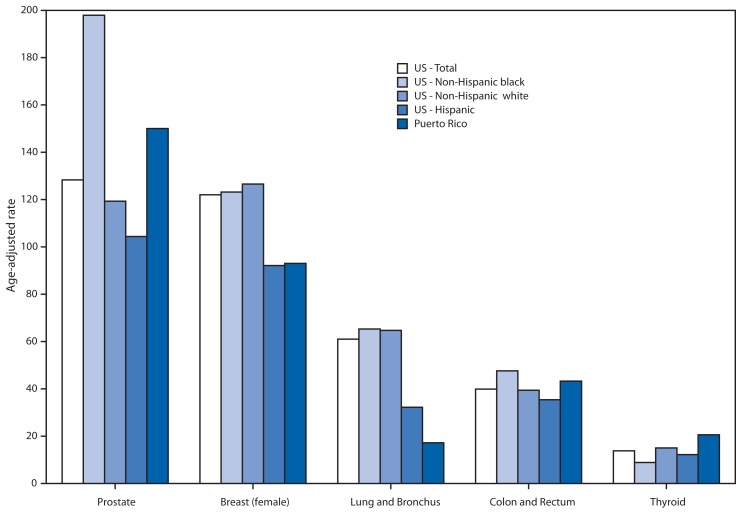
Age-adjusted rates* of invasive cancer^†^ incidence by selected primary cancer site, geographic location, race and ethnicity — National Program of Cancer Registries and the Surveillance, Epidemiology, and End Results program^§^, Puerto Rico and United States, 2011 * Incidence rates are per 100,000 persons and are age-adjusted to the 2000 U.S. standard population. ^†^ Excludes basal and squamous cell carcinomas of the skin except when these occur on the skin of the genital organs, and in situ cancers except urinary bladder. ^§^ Compiled from cancer registries that meet the data-quality criteria for all invasive cancer sites combined (representing 99% of the U.S. population).

**FIGURE 2:**
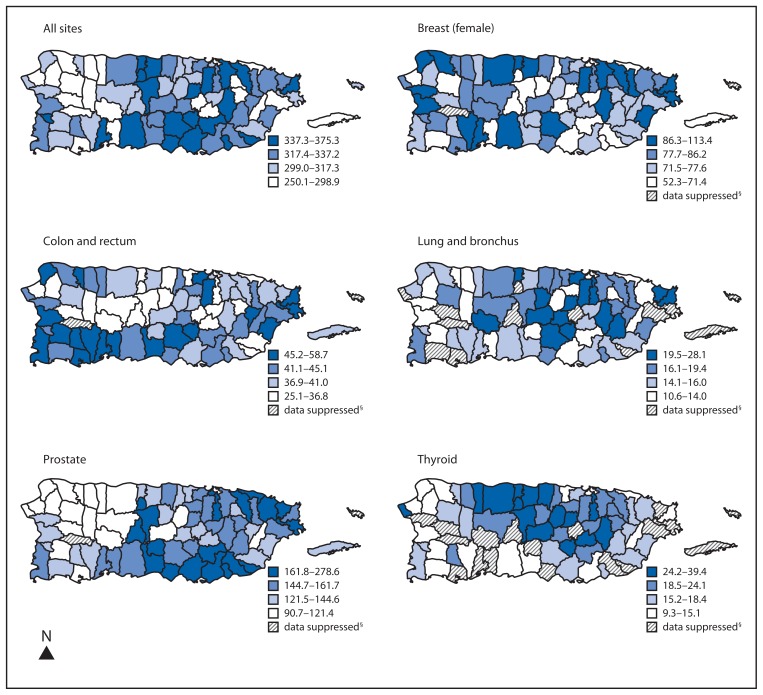
Age-adjusted incidence rates* of invasive cancer^†^ by selected primary cancer sites and municipality – National Program of Cancer Registries, Puerto Rico, 2007–2011 * Incidence rates are per 100,000 persons and are age-adjusted to the 2000 U.S. standard population. ^†^ Excludes basal and squamous cell carcinomas of the skin except when these occur on the skin of the genital organs, and in situ cancers except urinary bladder. ^§^ Data not given because <16 cases were reported.

**TABLE:** Age-adjusted rates* and numbers† of cancer incidence by sex, selected primary sites, and age group — National Program of Cancer Registries (NPCR), Puerto Rico, 2007–2011

Characteristic	Incidence					

	Overall		Males		Females	
		
	Rate	No.	Rate	No.	Rate	No.
**All cancer sites combined**	**329.7**	**68,312**	**395.1**	**37,068**	**281.3**	**31,244**
Brain and other nervous system	4.7	925	5.2	477	4.2	448
Breast (female)	NA	9,389	NA	NA	84.2	9,389
Cervix uteri	NA	1,215	NA	NA	12.2	1,215
Colon and rectum	42.6	8,891	52.5	4,880	34.8	4,011
Corpus and uterus, NOS	NA	2,332	NA	NA	20.5	2,332
Esophagus	3.7	776	6.6	620	1.3	156
Hodgkin lymphoma	2.5	481	3.0	267	2.1	214
Kaposi sarcoma	0.7	121	1.2	103	0.2	18
Kidney and renal pelvis	6.9	1,429	9.7	914	4.5	515
Larynx	3.5	743	7.0	664	0.7	79
Leukemias	6.8	1,338	8.3	734	5.7	604
Liver and intrahepatic bile duct	7.8	1,660	12.2	1,157	4.3	503
Lung and bronchus	17.0	3,575	24.5	2,274	11.2	1,301
Melanomas of the skin	2.7	547	3.5	313	2.2	234
Mesothelioma	0.2	40	0.4	34	DS	DS
Myeloma	3.9	818	4.9	446	3.2	372
Non-Hodgkin lymphoma	12.2	2,479	13.9	1,269	10.8	1,210
Oral cavity and pharynx	9.6	2,008	15.9	1,505	4.5	503
Ovary	NA	795	NA	NA	7.2	795
Pancreas	6.3	1,313	7.1	664	5.6	649
Prostate	NA	14,725	152.1	14,725	NA	NA
Stomach	8.4	1,752	11.4	1,030	6.2	722
Testis	NA	282	3.3	282	NA	NA
Thyroid	18.9	3,601	7.1	637	29.2	2,964
Urinary bladder	10.7	2,198	18.0	1,613	5.0	585
**Age group**						
0–19 years	13.5	701	13.9	368	13.0	333
20–49 years	127.5	9,152	85.4	2,933	165.6	6,219
50–64 years	594.2	20,875	683.7	11,200	518.6	9,675
65–74 years	1281.4	19,481	1762.7	12,177	880.5	7,304
≥75 years	1596.9	18,103	2235.7	10,390	1149.0	7,713

**Abbreviations:** DS = data suppressed (<16 cases were reported in the category); NA = not available; NOS = not otherwise specified.

* Incidence rates are per 100,000 persons and are age-adjusted to the 2000 U.S. standard population.

† Excludes basal and squamous cell carcinomas of the skin, except when these occur on the skin of the genital organs, and in situ cancers, except urinary bladder.

## Footnotes

* Invasive cancers are all cancers excluding basal and squamous cell skin cancers except when these occur on the skin of the genital organs, and in situ cancers except in the urinary bladder.

† Cancer registries demonstrated that cancer incidence data were of high quality by meeting the six USCS publication criteria: 1) case ascertainment is ≥ 90% complete, 2) ≤ 5% of cases are ascertained solely on the basis of a death certificate, 3) ≤ 3% of cases are missing information on sex, 4) ≤ 3% of cases are missing information on age, 5) ≤ 5% of cases are missing information on race, and 6) ≥ 97% of the registry’s records passed a set of single-field and inter-field computerized edits that test the validity and logic of data components. Additional information is available at http://www.cdc.gov/cancer/npcr/uscs/index.htm.

§ Additional information available at: http://www.cdc.gov/cancer/npcr/uscs/technical_notes/criteria.htm

¶ The 2007**–**2009 Puerto Rico population estimates were abstracted from the file **“**2000–2010 Puerto Rico Characteristics Intercensal Population Estimates,” source: U.S. Census Bureau, Population Division; release date: October 2012. The 2010**–**2011 Puerto Rico population estimates were abstracted from the file “Annual Estimates of the Resident Population by Single Year of Age and Sex for the United States, States, and Puerto Rico Commonwealth: April 1, 2010 to July 1, 2012.” U.S. Census Bureau, Population Division, release date June 2013.

** Annual estimates of the resident population by sex for Puerto Rico municipalities were provided by the U.S. Census Bureau, Population Division using the Vintage 2013 and 2000**–**2010 intercensal estimates series.

†† Additional information available at: http://ftp.cdc.gov/pub/Publications/Cancer/ccc/puerto_rico_ccc_plan_2008_2012.pdf.
